# Afatinib-induced Interstitial Lung Disease Successfully Treated with Corticosteroids: Case Report and Review of the Literature

**DOI:** 10.7759/cureus.2805

**Published:** 2018-06-14

**Authors:** Amanda L Jobe, Kyle T Ahonen, Victoria Poplin, Sunitha Rao, Ghulam Rehman Mohyuddin

**Affiliations:** 1 Internal Medicine, Kansas University Medical Center, Kansas City, USA; 2 Psychiatry, Kansas University Medical Center, Kansas City, USA; 3 Internal Medicine, Kansas Univeristy, Kansas City, USA; 4 Internal Medicine, Dwight D Eisenhower Va Medical Center, Leavenworth , USA; 5 Internal Medicine, Kansas University Medical Center, Kansas City, USA

**Keywords:** lung cancer, pneumonitis

## Abstract

The first-line treatment for advanced epidermal growth factor receptor (EGFR) mutation positive non-small cell lung cancer (NSCLC) includes the use of afatinib and other EGFR tyrosine kinase inhibitors (EGFR-TKIs). While generally well tolerated, a small subset of patients will develop drug-induced interstitial lung disease (ILD) which could lead to drug discontinuation or even death.

A 58-year-old female with stage IV NSCLC treated with afatinib presented with dyspnea and rapidly progressive hypoxemia. Imaging of the lungs demonstrated ground glass opacities. Infectious workup was unrevealing, and since drug-induced ILD was suspected early on presentation, high dose corticosteroids were initiated leading to clinical improvement.

While the incidence of afatinib-induced ILD is rare, the consequences may be serious and potentially fatal. The presentation is often non-specific and may mimic other common respiratory pathologies making the diagnosis challenging. If therapeutic measures such as corticosteroids are initiated promptly, they can be life-saving.

## Introduction

Lung cancer remains the leading cause of cancer-related deaths worldwide. Non-small cell lung cancer (NSCLC) accounts for about 85% of lung cancer cases, and between 10 and 30% of patients carry the epidermal growth factor receptor (EGFR) mutation [1,2]. The first-line treatment for advanced EGFR mutation positive NSCLC includes the use of EGFR tyrosine kinase inhibitors (EGFR-TKIs) [3-5]. Afatinib has been shown to have improved outcomes over gefitinib and erlotinib due to the development of resistance to the first generation TKIs [4, 6]. While the EGFR-TKIs are generally well tolerated, a small subset of patients will develop drug-induced interstitial lung disease (ILD) which could lead to drug discontinuation or even death [3, 5, 7].

## Case presentation

A 58-year-old female with a history of stage IV NSCLC of her right lung presented to the emergency department with complaints of shortness of breath for four days.

Her lung cancer was discovered 3.5 years earlier as a 4 x 5.3 cm right upper lung cavitary mass on routine lung computed tomography (CT) screening (T3N2M0, stage IIIA at diagnosis). She did have a long-standing history of tobacco use.

She underwent seven months of treatment with radiation and remained disease free until about two years later, when a sample from a pleural effusion confirmed lung adenocarcinoma. Molecular studies were negative for anaplastic lymphoma kinase (ALK), ROS-1, and programmed death ligand 1 (PD-L1) but positive for EGFR. She underwent one cycle of carboplatin and paclitaxel prior to finding this mutation. She was then switched to erlotinib. Six months later, CT showed the progression of the disease, so she was switched to afatinib at a dose of 40 mg daily.

**Figure 1:**
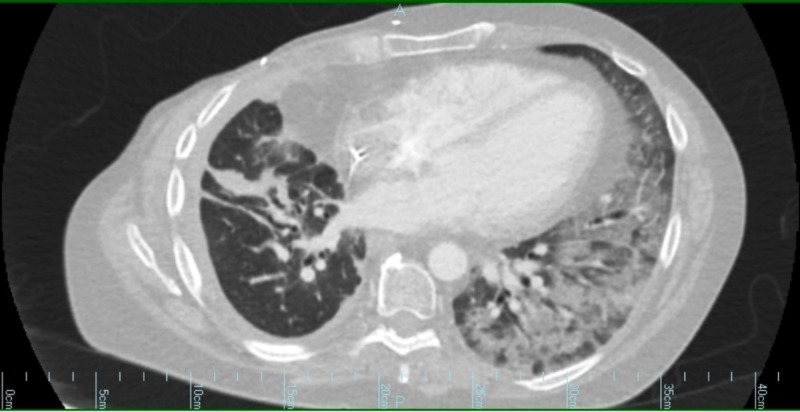
Computed tomography (CT) scan of the chest. A CT scan of the chest demonstrating consolidation and ground glass opacities predominately involving the left lung.

**Figure 2:**
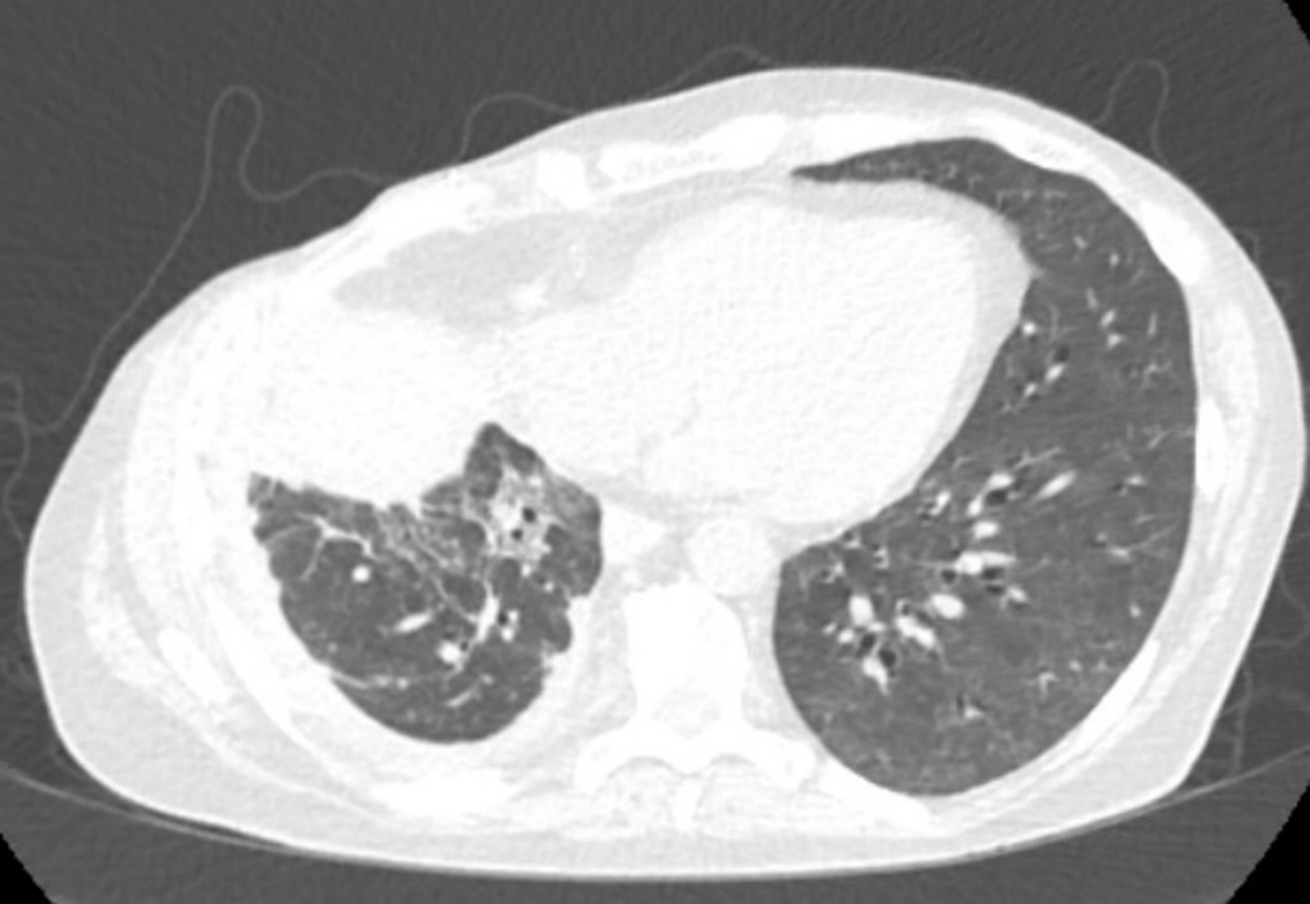
Subsequent computed tomography (CT) scan of the chest. Subsequent CT scan of the chest showing marked improvement in the ground glass opacities in the left lung, although the primary lung mass has increased in size.

She presented to the emergency department one month after starting afatinib. Initial evaluation was significant for a new two-liter oxygen requirement. Her blood work was unrevealing other than a mild non-gap metabolic acidosis secondary to chronic diarrhea. She was admitted and started on empiric broad-spectrum antibiotics. Afatinib was held at admission. CT chest with contrast was obtained that showed no pulmonary embolism but did demonstrate significantly increased ground glass opacities in the left lung, a right perihilar mass unchanged from prior scans, a right pleural effusion, and enlarged main pulmonary arteries (Figure 1). Two days after admission, she developed profound hypoxemia requiring escalation to a non-rebreather to maintain oxygenation. A bronchoscopy revealed no endobronchial lesions, and the sample obtained showed scant white blood cells (70% monocytes, 10% polys) with no organisms. Despite completing a seven-day course of broad-spectrum antibiotics, a thorough infectious workup (including bacterial cultures, respiratory viral panel and procalcitonin) was unrevealing. The patient was started on a daily dose of 500 mg methylprednisolone on hospital day three due to a suspicion for afatinib-induced pneumonitis. After one week, the patient’s oxygen requirement gradually improved allowing for a decrease in steroid dosing. Unfortunately, she then suffered a bowel perforation (presumed to be unrelated to her pulmonary presentation) requiring sub-total colectomy with end ileostomy. She was discharged to a skilled nursing facility subsequently. At her one month post-discharge follow-up, she was off steroids and saturating well on two liters of supplemental oxygen. A follow-up CT scan obtained four months later showed marked improvement of the pneumonitis, although the primary lung cancer had increased in size (Figure 2).

## Discussion

Multiple meta-analyses have evaluated the risk of treatment-related toxicities associated with the EGFR-TKI’s [3, 5, 7] with an estimated incidence of all-grade ILD of 1.6%, high-grade ILD (≥ grade 3) of 0.9%, and a mortality rate of 13% [3]. Onset is usually within three months of therapy, however, the risk of developing ILD has not shown to be associated with the treatment duration of EGFR-TKI's [3]. Analyses have failed to show any significant difference in the rate of ILD between gefitinib, erlotinib, or afatinib [3, 7].

The mechanism for drug-induced ILD is not well understood but it has been suggested that EGFR inhibits a receptor that promotes alveolar wall repair [3]. Patients typically present with non-specific signs and symptoms including low-grade fever, cough, shortness of breath, and/or hypoxemia, and the degree of severity may vary dramatically among patients. The diagnosis can be challenging, as the onset is unpredictable, patients may display multiple different types of radiographic injury patterns, and the clinical picture may mimic other pathologies such as infection or metastasis [8]. Treatment includes drug discontinuation, corticosteroid therapy, and supportive measures such as supplemental oxygenation and potentially mechanical ventilation [3, 8]. In 2017, Fujita et al. demonstrated the use of corticosteroids as a potential life-saving measure in a patient specifically with afatinib-induced ILD, and corticosteroids were also life-saving for our patient. Therefore, we propose steroids be used in patients with afatinib-induced ILD [9].

## Conclusions

While the incidence of EGFR-TKI-induced ILD is rare, the consequences may be serious and potentially fatal. Providers must be astutely aware of this in patients presenting with respiratory complaints, as the presentation is often non-specific and may mimic other common respiratory pathologies. If therapeutic measures such as corticosteroids are initiated promptly as in our case, they can be life-saving.
